# Association of Diet- and Health-Related Beliefs with Restrictive Diets Without Medical Indication in a Self-Selected Online Sample of Adults in Romania: A Cross-Sectional Study

**DOI:** 10.3390/nu18162685

**Published:** 2026-08-17

**Authors:** Nina Ciuciuc, Codruța Alina Popescu, Alexandra-Ioana Roșioară, Giulia-Ecaterina Stan, Anca Lucia Pop, Ovidiu Nicolae Penes, Valentin Nicolae Varlas, Alexandra Maria Moldovan, Bogdana Adriana Năsui

**Affiliations:** 1 Department of Community Medicine, “Iuliu Hațieganu” University of Medicine and Pharmacy, 400349 Cluj-Napoca, Romania; nina.ciuciuc@umfcluj.ro (N.C.); alexandra.rosioara@umfcluj.ro (A.-I.R.); ale_moldovan2002@yahoo.com (A.M.M.); adriana.nasui@umfcluj.ro (B.A.N.); 2 Research Center in Preventive Medicine, Health Promotion and Sustainable Development, “Iuliu Hațieganu” University of Medicine and Pharmacy, 400349 Cluj-Napoca, Romania; 3 Department of Human Sciences, “Iuliu Hațieganu” University of Medicine and Pharmacy, 6 Louis Pasteur Street, 400349 Cluj-Napoca, Romania; 4 Data Analyst, Ernst&Young SRL, 400002 Cluj-Napoca, Romania; giulia.stan10@gmail.com; 5 Department of Clinical Laboratory, Food Safety, “Carol Davila” University of Medicine and Pharmacy, Traian Vuia Street, 020945 Bucharest, Romania; anca.pop@umfcd.ro; 6 Department of General Medicine, “Carol Davila” University of Medicine and Pharmacy, 37 Dionisie Lupu Street, 020021 Bucharest, Romania; ovidiu.penes@umfcd.ro; 7 Department of Obstetrics and Gynaecology, Filantropia Clinical Hospital, 050474 Bucharest, Romania; valentin.varlas@umfcd.ro

**Keywords:** restrictive diets, health beliefs, nutrition knowledge, food vigilance, social networks, eating behavior, public health

## Abstract

Background: The adoption of restrictive diets without medical indication is increasing, but the cognitive factors associated with this dietary behavior remain insufficiently understood. The aim of this study was to examine the proportion of participants following restrictive diets in a self-selected online sample of adults living in Romania and to analyze the associations of nutrition knowledge, beliefs regarding the relationship between diet and health, food vigilance, and social media use with the adoption of restrictive diets without medical indication. Methods: This cross-sectional observational study was conducted online in a self-selected sample of 766 adults living in Romania. Participants were classified into three groups: no restrictive diet, medically indicated restrictive diet, and restrictive diet without medical indication. Three composite scores were constructed: Restrictive Health Beliefs, Food Vigilance, and Nutrition Knowledge. Differences between groups were assessed using non-parametric tests and χ^2^ tests, and factors associated with the adoption of restrictive diets were analyzed using multinomial logistic regression and multiple linear regression. Results: Among the participants, 30.9% reported following a restrictive diet, and 13.1% adopted one without medical indication. Participants who followed restrictive diets scored higher for Restrictive Health Beliefs and Food Vigilance but not Nutrition Knowledge. In the multivariate analysis, Restrictive Health Beliefs showed the strongest independent association with restrictive diets without medical indication. Food Vigilance was only associated with medically indicated restrictive diets (*p* < 0.001), suggesting an adaptive role in a therapeutic context. Nutrition knowledge and social media use were not independently associated with restrictive diets without medical indication, although social media use was associated with higher levels of Restrictive Health Beliefs. Conclusions: The results suggest that the adoption of restrictive diets without medical indication is more closely associated with beliefs regarding the relationship between diet and health than with the level of nutrition knowledge. These findings support the development of nutrition education interventions that, in addition to conveying evidence-based information, promote the development of critical thinking and the correction of insufficiently scientifically substantiated dietary beliefs.

## 1. Introduction

Restrictive diets have become increasingly common and are adopted not only in cases of food intolerance, allergy, or other medical conditions but also as personal choices related to perceptions of healthy eating, weight control, disease prevention, or general health improvement [1,2,3,4]. The distinction between medically indicated and non-medically indicated dietary restrictions is important because the same dietary exclusion may have different nutritional and clinical implications depending on the context and the reason for its adoption. When restrictions are adopted without a diagnosis or medical recommendation, they may reflect dietary beliefs, perceptions of risk, personal preferences, or the influence of informal sources of information [5,6].

For individuals with a confirmed diagnosis, avoiding gluten, lactose, or other food components may be necessary and may require medical and nutritional monitoring [1,7,8]. In the absence of a clear medical indication, however, complete exclusion of foods or food groups may reduce dietary diversity and contribute to inadequate intake of fiber, calcium, vitamin D, or other nutrients when appropriate alternatives are not used [2,6,8,9,10,11]. General health recommendations may also be interpreted rigidly, leading to the avoidance of foods perceived as inflammatory, acidic, unnatural, or unsafe, even when complete exclusion is not clinically supported [3,4,12,13].

Nutrition knowledge may help individuals distinguish between evidence-based recommendations, medical advice, and insufficiently supported dietary claims. However, the relationship between nutrition knowledge and eating behavior is not necessarily direct. Nutrition knowledge represents one component of broader nutrition literacy, which also includes the ability to select, interpret, evaluate, and apply food-related information appropriately [14,15]. Individuals may possess general knowledge about healthy eating while interpreting selected information through pre-existing beliefs about the relationship between food and health. Consequently, certain dietary restrictions may be perceived as inherently beneficial even in the absence of a medical indication [3,4,14].

Diet-related health beliefs and food vigilance may also be relevant to restrictive dietary behavior. The perception that specific foods are harmful, inflammatory, unnatural, or unsafe may be associated with avoiding them, while labels such as “gluten-free,” “lactose-free,” “natural,” “clean,” or “healthier” may shape consumers’ perceptions independently of the actual nutritional value of a product [3,4,16,17]. Studies on “free-from” products indicate that they are also consumed by individuals without a diagnosis or clear medical recommendation, often because they are perceived as healthier, more natural, or safer [3,4]. Food vigilance, including attention to food quality, composition, labels, and perceived health effects, may support appropriate dietary management in a therapeutic context. However, when accompanied by rigid beliefs, fear, or simplified distinctions between “good” and “bad” foods, it may also be associated with unnecessary dietary restrictions [14,16,18,19].

The digital environment may amplify these processes. Social media, video platforms, blogs, and online communities have become important sources of nutrition information, although the quality and scientific accuracy of the available content vary considerably [5,20,21]. Evidence-based recommendations are often presented alongside personal experiences, promotional content, and simplified claims regarding detoxification, inflammation, food intolerances, or foods considered harmful [5,21]. In this context, nutrition information encountered on social media may contribute to shaping beliefs about the benefits or risks associated with certain foods. People who adopt restrictive diets without medical advice may also lack appropriate monitoring, counseling, or nutritionally equivalent alternatives, potentially increasing the risk of dietary imbalances [2,6,8,10].

Although previous studies have examined nutrition knowledge, diet-related health beliefs, food vigilance, and the influence of online information, these factors have generally been investigated separately. Few studies have assessed their relative associations with restrictive dietary behavior while distinguishing between medically indicated restrictive diets and those adopted without a medical indication. Consequently, it remains unclear whether these two forms of dietary restriction are associated with similar or different patterns of nutrition knowledge, health beliefs, food vigilance, and social media use. To address this gap, we distinguished between medically indicated restrictive diets and those adopted without a medical indication, enabling factors associated with therapeutic dietary restrictions and those adopted voluntarily in the absence of a medical indication to be examined separately.

The aim of this study was to examine the proportion of participants following restrictive diets in a self-selected online sample of adults living in Romania and to assess the associations of nutrition knowledge, beliefs about the relationship between diet and health, food vigilance, and social media use with restrictive dietary behavior. More specifically, we sought to examine whether these factors were independently associated with medically indicated or non-medically indicated restrictive diets and to identify which factor showed the strongest independent association with restrictive diets adopted without a medical indication. Based on the existing literature, we hypothesized that individuals following restrictive diets without a medical indication would report stronger beliefs about the relationship between diet and health than those not following such diets, regardless of their level of nutrition knowledge. We further hypothesized that nutrition knowledge would show a weaker association with restrictive dietary behavior than diet-related health beliefs and that social media use and food vigilance would be associated with stronger beliefs about the relationship between diet and health.

## 2. Materials and Methods

### 2.1. Study Design and Participants

This was an observational, cross-sectional study conducted using a self-administered online questionnaire among adults aged ≥18 years living in Romania. Questionnaires with incomplete responses, those from individuals under 18 years of age, and those with inconsistent or missing information for the study’s main variables were excluded. No formal a priori sample size calculation was performed. All eligible and valid questionnaires collected during the predefined recruitment period were included in the analysis. The final sample size allowed us to perform the comparative analyses and construct the multivariable models we had planned. However, the absence of an a priori sample size calculation should be considered when interpreting the precision and generalizability of the estimates.

Data were collected using Google Forms between May and June of 2026. All participants were apprised of the purpose of the study and provided their informed consent by voluntarily and anonymously completing the questionnaire; a separate informed consent form was not required.

The reporting of this cross-sectional study was guided by the Strengthening the Reporting of Observational Studies in Epidemiology (STROBE) Statement [22].

### 2.2. Recruitment Strategy and Participant Flow

Participants were recruited through the distribution of the questionnaire in social media groups and online communities focused on nutrition, restrictive diets, food intolerances, and healthy lifestyles. To increase the visibility of the study, the invitation to participate was also shared through public accounts with large audiences that were relevant to health, nutrition, and public communication. A convenience sampling strategy was used in the online environment. Recruitment through social media enabled rapid access to individuals interested in nutrition, restrictive diets, and healthy lifestyles, a method frequently used in online studies [23].

To ensure methodological transparency, the recruitment process was traced from the potential online audience to the final number of valid questionnaires included in the analysis. The potential audience was estimated at approximately 300,000 individuals, based on the cumulative number of followers of the public accounts and members of the online communities through which the invitation was distributed. This figure represents the theoretical maximum audience of the distribution channels and not the actual number of individuals who viewed the invitation.

The number of individuals who viewed the invitation or accessed the survey link could not be determined precisely because complete analytics were not available for all distribution channels; as a result, view and participation rates could not be calculated. In accordance with the CHERRIES recommendations, these stages were reported as “not recorded” [24].

Rates of response to online surveys may vary according to the target population, study topic, and distribution method [25,26].

The participant recruitment process consisted of five stages:

1. Potential audience—approximately 300,000 individuals, estimated on the basis of the cumulative audience of the distribution channels.
2. Viewing the invitation—not recorded.
3. Clicking the survey link—not recorded.
4. Completing the survey—in total, there were 779 completed and submitted surveys.
5. Questionnaire validation—of the 779 completed and submitted questionnaires, 13 were excluded: 6 because of incomplete responses, 5 because the respondents were under 18 years of age, and 2 due to missing or inconsistent information for the main study variables. A total of 766 valid questionnaires were included in the final analysis.

**Figure 1:**
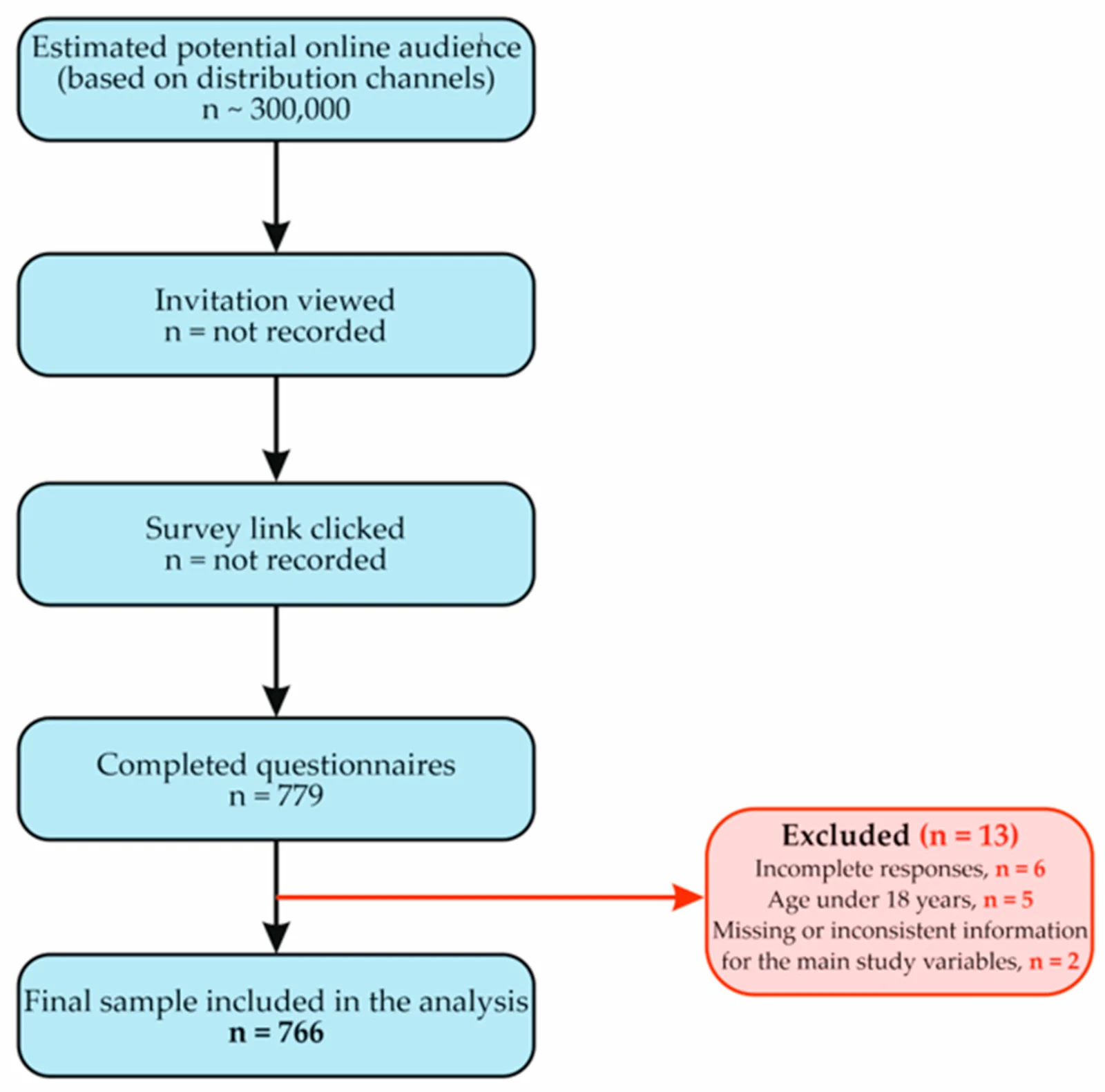
Flow diagram of online recruitment and participant inclusion process.

The entire process, from recruitment to the inclusion of participants in the final analysis, is illustrated in Figure 1.

To reduce potential classification and data-quality bias, questionnaires with incomplete, inconsistent, or missing information for the main study variables were excluded before analysis.

### 2.3. Study Instrument and Pilot Testing

Data were collected online using a questionnaire developed specifically for this study. The questionnaire included 48 items grouped into the following domains: demographic data, nutritional status, adherence to a restrictive diet, motivations and perceptions related to the adopted diet, attitudes toward diet and health, general nutrition knowledge, and practices associated with adopting a restrictive diet. The full questionnaire is provided as Supplementary File S1. To assess attitudes toward nutrition and health, items selected and adapted from the Health and Taste Attitude Scales (HTAS) were used. The HTAS examines concern regarding healthy eating, preference for natural products, and the importance of taste in food choices [27,28]. In this study, items relevant to interest in healthy eating, the perception of the healthiness or naturalness of foods, reading labels, and avoiding food components perceived as having adverse health effects were used.

Nutrition knowledge was assessed using items derived from the structure of and topics covered by the General Nutrition Knowledge Questionnaire (GNKQ) and the General Nutrition Knowledge Questionnaire-Revised (GNKQ-R), which include nutritional recommendations, dietary sources of nutrients, healthy food choices, and the relationship between diet, disease, and weight [29,30]. In this study, the items were adapted to the study objectives, with an emphasis on a balanced diet, dietary sources of nutrients, gluten, lactose, and the risks of eliminating certain food groups without medical advice.

The items selected from the original English-language instruments were translated into Romanian, culturally adapted, and subsequently retranslated into English (forward–backward translation) to ensure linguistic and conceptual equivalence, in accordance with methodological recommendations regarding the cross-cultural adaptation of psychometric instruments.

The questionnaire did not use the full versions of the HTAS and GNKQ/GNKQ-R but rather items selected from these instruments, supplemented with specific questions to meet this study’s objectives. Consequently, the scores derived from these items were interpreted as exploratory indicators of the nutritional attitudes, beliefs, and knowledge being assessed.

The questionnaire also included questions regarding types of dietary restrictions, the existence of a medical recommendation, the reported diagnosis, the influence of social media on food choices, and other practices associated with restrictive diets, which were necessary for classifying the participants into the three groups analyzed.

The questionnaire was pilot-tested on a sample of 30 respondents to evaluate the clarity of the items, the consistency of the form, and the feasibility of completion—a procedure recommended for studies assessing nutrition knowledge, attitudes, and practices [31]. The mean questionnaire completion time was approximately 20–25 min, and the internal consistency of the questionnaire was excellent (Cronbach’s α = 0.88).

### 2.4. Operational Classification

In this study, participants were classified into three groups based on their responses to questions regarding the existence of a restrictive diet, the presence of a medical recommendation, and the existence of a relevant reported diagnosis. A restrictive diet was defined as the intentional avoidance or exclusion of one or more foods, food components, or food groups (gluten-free, lactose-free, vegan, or other diets based on the elimination of certain foods) [32]. Participants who did not report a restrictive diet were included in the group without a restrictive diet. Participants who reported a restrictive diet associated with a medical recommendation and/or a self-reported diagnosis compatible with the dietary restriction were classified into the medically justified restrictive diet group.

Participants who reported a restrictive diet in the absence of a medical recommendation and a relevant self-reported diagnosis were classified into the non-medically indicated restrictive diet group. This operational classification is consistent with the literature on elimination diets and the consumption of “free-from” products, which distinguishes between medically recommended dietary restrictions and food avoidance behaviors adopted without a clear medical justification [3,4,32].

The specific type of diet reported by the participant was not used to assign participants to the three operational groups because the same dietary restriction could be followed with or without a medical recommendation or justification. The diet types reported were analyzed separately for descriptive purposes.

### 2.5. Sociodemographic, Anthropometric, and Diet-Related Variables

- The demographic data collected were age, sex, and place of residence (urban/rural).
- Nutritional status was assessed using body mass index (BMI), calculated based on self-reported weight and height. Participants were classified into BMI categories according to the World Health Organization classification [33] as follows:
  1. underweight (below 18.5 kg/m^2^); 2. normal weight (18.5–24.9 kg/m^2^); 3. overweight (25–29.9 kg/m^2^); 4. class I obesity (30–34.9 kg/m^2^); 5. class II obesity (35–39.9 kg/m^2^); 6. class III obesity (40 kg/m^2^ or more).
- Variables related to dietary restriction included adherence to a special diet, the type of diet followed, the duration of adherence, the existence of a medical recommendation, and the presence of a reported diagnosis that could justify the dietary restriction. This information was subsequently used in the operational classification (Section 2.4). Participants could report one or more types of restrictive diet. Responses that did not correspond to the predefined categories were recorded under “Other diets.” Because the questionnaire did not request further details regarding the specific type of diet included in this category, a more detailed breakdown was not possible. The “Other diets” category was used exclusively for descriptive purposes and did not influence the participants’ classification into the three operational groups.
- Beliefs and perceptions associated with restrictive diets were assessed through questions regarding the participants’ perceptions of foods or food components such as gluten, lactose, histamine, acidic foods, and other products considered to have negative effects on health. Perceptions regarding “free-from” products and the perception that eliminating certain food components may be associated with general health benefits or weight control were also investigated.
- Food vigilance was assessed through items regarding attention to food quality and composition, reading labels, avoiding additives or preservatives, a preference for natural foods, and concern about the health effects of diet. The influence of social media on dietary decisions was assessed using an item regarding the use of social media as a source of nutrition information for food choices.
- Practices associated with restrictive diets were assessed through questions regarding consultation with a physician or nutritionist before starting the diet and attitudes toward recommending a restrictive diet to others without prior medical testing or professional advice.

For statistical analysis, age and BMI were analyzed as continuous variables. Variables regarding the special diet, medical recommendation, and self-reported diagnosis were used according to the operational classification described above. Items regarding restrictive dietary beliefs, dietary vigilance, and nutrition knowledge were used to construct composite scores (Section 2.6).

### 2.6. Construction of Composite Scores

For the main analysis, three composite scores were constructed: the Restrictive Health Beliefs Score, the Food Vigilance Score, and the Nutrition Knowledge Score.

The Restrictive Health Beliefs Score assessed beliefs regarding the perceived negative effects of certain foods or food components on health, as well as the perception that “free-from” products are safer, more natural, or more beneficial to health. The score was calculated as the average of responses to 9 items regarding gluten, lactose, histamine, acidic foods, iodized salt, and the perceived benefits of eliminating certain food groups. The score had a theoretical range of 1–5, with higher values indicating stronger restrictive dietary beliefs.

The Food Vigilance Score assessed the attention paid to diet and food choices, including interest in food quality and composition, reading labels, avoiding additives and preservatives, a preference for natural foods, and concern about the impact of diet on health. The score was calculated as the average of responses to 8 items. The score had a theoretical range of 1–5, with higher values indicating a higher level of food vigilance.

The Nutrition Knowledge Score assessed participants’ general nutrition knowledge and their ability to identify correct or incorrect information regarding a balanced diet, dietary sources of nutrients, and the effects of eliminating certain food groups without medical advice. The score included 17 nutrition knowledge questions. Correct answers were coded as 1 and incorrect answers as 0; the total score was calculated by summing the correct answers (range: 0–17 points).

The internal consistency of the scores constructed from Likert-type items was assessed using Cronbach’s α coefficient. Since the scores were constructed for the specific purpose of this study and do not represent full versions of validated instruments, the resulting Cronbach’s α was interpreted as exploratory, and the scores were used as indicators of restrictive dietary beliefs, dietary vigilance, and nutrition knowledge assessed in this context.

### 2.7. Statistical Analysis

The data were initially entered into a Microsoft Excel database and subsequently analyzed statistically using Jamovi v2.7.22. Categorical variables were expressed as absolute frequencies and percentages, and continuous variables as means and standard deviations or medians and interquartile ranges, depending on the data distribution. The distribution of continuous variables was assessed using the Shapiro–Wilk test.

The internal consistency of the scores derived from Likert-type items—namely, the Restrictive Health Beliefs Score and the Food Vigilance Score—was assessed using Cronbach’s α and interpreted exploratorily given the specific nature of the scores developed for this study.

Differences among the three operational groups—no restrictive diet, medically justified restrictive diet, and restrictive diet without medical indication—were analyzed using χ^2^ tests, Fisher’s exact test, or Monte Carlo simulations, as appropriate, for categorical variables and using the Kruskal–Wallis test for continuous or ordinal variables. When the Kruskal–Wallis test indicated significant differences, post hoc comparisons were performed using the Dunn test with Bonferroni correction. For associations between categorical variables, Cramer’s V was reported, and for nonparametric comparisons, effect sizes (ε^2^) were reported, as appropriate.

The associations between composite scores, age, and BMI were assessed using Spearman’s correlation coefficient. Differences in scores based on socioeconomic background and education level were analyzed using the Mann–Whitney and Kruskal–Wallis tests, respectively.

To identify factors associated with membership in the three diet groups (no restrictive diet, medically justified diet, and diet without medical indication), a multinomial logistic regression model was used. The reference category for the model was participants not following a restrictive diet. The predictors included in the model were the Restrictive Health Beliefs Score, the Food Vigilance Score, the Nutrition Knowledge Score, the extent to which participants reported being influenced by social media in their food choices, age, sex, BMI, and education level. The results were reported as odds ratios (OR), 95% confidence intervals, and *p*-values.

To identify factors associated with the Restrictive Health Beliefs Score, a multiple linear regression model was constructed. The included predictors were the Food Vigilance Score, the Nutrition Knowledge Score, the influence of social media, sex, age, BMI, socioeconomic background, and education level. The results were reported as standardized β coefficients and *p*-values. Statistical significance was set at *p* < 0.05.

No imputation of missing data was performed. Questionnaires with missing or inconsistent information for the main variables required for participant classification and the primary analyses were excluded before analysis. A small number of missing values remained for secondary descriptive variables that were not required for participant classification or for the main analytical objectives of the study. In these cases, descriptive analyses were based on the available responses, and the corresponding denominator was reported where appropriate.

## 3. Results

### 3.1. Sample Characteristics

The final sample included 766 respondents. The mean age of the participants was 40.9 ± 11.1 years. The most well-represented age groups were 35–44 years and 45–54 years, indicating a predominance of middle-aged adults in the sample.

The sample was predominantly female and urban. Most respondents were from urban areas (87.3%), and more than half were female (56.3%). The educational level of the sample was high, with over 86% of respondents reporting a university degree (bachelor’s, master’s, or doctorate).

The average body mass index (BMI) was 26.7 ± 5.7 kg/m^2^. According to the WHO classification, the largest proportion of participants were of normal weight (37.9%), followed by overweight (32.6%). Class I obesity was observed in 17.9% of respondents, while Class II and III obesity were present in 4.3% and 2.9%, respectively. Underweight participants accounted for 4.4% of the total sample (Table 1).

### 3.2. Characteristics of Restrictive Diets in the Study Sample

Of the total number of participants, 237 (30.9%) reported following a special or restrictive diet, while 529 (69.1%) reported no dietary restrictions. A total of 102 participants (13.3%) reported having a diagnosed food intolerance, with gluten intolerance being the most commonly reported, followed by lactose intolerance and histamine intolerance. According to the presence or absence of a medical recommendation, 17.9% of respondents reported following a medically recommended diet, while 13.1% adopted a restrictive diet without a medical recommendation. Among the specific predefined restrictive diet categories, gluten-free and lactose-free diets were the most frequently reported, while low-histamine, iodine-free, and alkaline diets were rare. The heterogeneous “Other diets” category included 117 reports corresponding to diets not covered by the predefined response options. Most participants following a diet reported having done so for more than six months (Table 2).

### 3.3. Characteristics of the Composite Scores

Three composite scores were constructed to assess restrictive health beliefs (Restrictive Health Beliefs Score), food vigilance (Food Vigilance Score), and nutrition knowledge (Nutrition Knowledge Score) (Table 3). The Restrictive Health Beliefs Score had the lowest mean value (1.84 ± 0.84), suggesting a relatively low level of restrictive beliefs regarding the elimination of certain foods or food components. The Food Vigilance Score had a mean value of 3.46 ± 0.67, indicating a moderate to high level of attention paid to diet and food composition. The Nutrition Knowledge Score had a mean value of 14.2 ± 1.77 points, suggesting a relatively high level of nutrition knowledge.

### 3.4. Comparison of Composite Scores Among Participants on a Non-Restrictive Diet, a Medically Indicated Diet, and a Diet Without Medical Indication

A comparison of the three groups revealed significant differences in the Restrictive Health Beliefs Score and the Food Vigilance Score (both *p* < 0.001), while the Nutrition Knowledge Score did not differ significantly across groups (*p* = 0.293) (Table 4). The Restrictive Health Beliefs Score was the highest in the group of participants following a medically justified diet (Md = 2.50; IQR = 1.25), followed by the participants adhering to a restrictive diet without medical indication (Md = 2.00; IQR = 1.50). The lowest values were observed among participants not following a restrictive diet (Md = 1.38; IQR = 1.13). Post hoc analyses revealed significant differences among all three groups (all *p* < 0.001).

A similar pattern was observed for the Food Vigilance Score, with values increasing progressively from participants not on a restrictive diet to those following a diet without medical indication, with the highest values observed in the group of participants following a medically justified diet. Post hoc analyses indicated significant differences among all three groups, including between participants on a diet without medical indication and those not on a restrictive diet (*p* = 0.047).

In contrast, the Nutrition Knowledge Score showed similar values across all three groups, with medians ranging from 14 to 15 points.

### 3.5. Sociodemographic and Anthropometric Characteristics of Participants by Type of Restrictive Diet

A comparison of the sociodemographic and anthropometric characteristics of the participants across the three diet groups revealed statistically significant differences in sex distribution, place of residence, and BMI but not in age or education level (Table 5). The sex distribution differed significantly across the three groups (χ^2^ = 63.8, *df* = 4, *p* < 0.001); women represented 85.4% of participants in the medically indicated diet group, 61.0% of those following a diet without medical indication, and 47.8% of those without a restrictive diet, while men represented 14.6%, 39.0%, and 51.8% of these groups, respectively. The two participants who preferred not to indicate their sex were both in the group without a restrictive diet. Participants from urban areas predominated in all three groups; the medically indicated diet group included the highest proportion of participants from rural areas (21.2%). BMI differed significantly among the groups (*p* = 0.014), with lower median values in the medically indicated diet group than in the group not following a restrictive diet. Age and education level did not differ significantly between groups (*p* > 0.05).

### 3.6. Relationships Among Restrictive Health Beliefs, Food Vigilance, Nutrition Knowledge, and Participant Characteristics

The strongest correlation observed was between the Restrictive Health Beliefs Score and the Food Vigilance Score (ρ = 0.378, *p* < 0.001), suggesting that participants with stronger beliefs regarding the benefits of restrictive diets tended to pay closer attention to their diet and the composition of their food (Table 6).

The Restrictive Health Beliefs Score was negatively correlated with the Nutrition Knowledge Score (ρ = −0.264, *p* < 0.001), indicating that participants with more pronounced restrictive beliefs had, on average, lower levels of nutrition knowledge.

The Food Vigilance Score was weakly positively correlated with age (ρ = 0.133, *p* < 0.001), suggesting slightly greater attention to diet among older participants.

Weak negative correlations were observed between BMI and the Restrictive Health Beliefs Score (ρ = −0.077, *p* = 0.034) and the Food Vigilance Score (ρ = −0.097, *p* = 0.007), respectively.

The influence of social media on food choices was positively correlated with the Restrictive Health Beliefs Score (ρ = 0.231, *p* < 0.001), suggesting that participants who reported drawing inspiration from social media more frequently tend to hold stronger beliefs regarding the benefits of restrictive diets.

### 3.7. Factors Associated with the Adoption of Restrictive Diets

The regression model identified sex, the Restrictive Health Beliefs Score, and the Food Vigilance Score as factors significantly associated with diet group membership. In contrast, age, BMI, and the Nutrition Knowledge Score were not significantly associated with the type of diet followed (Table 7). Compared to participants without a restrictive diet, those following a medically justified diet presented significantly higher Restrictive Health Beliefs Score and Food Vigilance Score values and were more frequently female. A one-point increase in the Restrictive Health Beliefs Score was associated with approximately 2.8-fold higher odds of following a medically justified diet (OR = 2.79), while a one-point increase in the Food Vigilance Score was associated with approximately 2.2-fold higher odds of membership in this group (OR = 2.25). In the case of restrictive diets without medical indication, the Restrictive Health Beliefs Score remained the only factor independently associated with group membership. A one-point increase in this score was associated with approximately 1.7-fold higher odds of following a restrictive diet without medical indication (OR = 1.68).

The Restrictive Health Beliefs Score showed the most consistent independent association with restrictive diet-group membership, while the Nutrition Knowledge Score was not independently associated with membership in either restrictive-diet group.

### 3.8. Factors Associated with Restrictive Dietary Beliefs

Multiple linear regression identified several factors independently associated with the Restrictive Health Beliefs Score (Table 8). The model was statistically significant and explained 28.6% of the variance in the Restrictive Health Beliefs Score (R^2^ = 0.286; *p* < 0.001).

The strongest association was observed between the Food Vigilance Score and the Restrictive Health Beliefs Score. Participants who reported higher levels of food vigilance and food composition had stronger restrictive health beliefs. Conversely, higher levels of nutrition knowledge were associated with lower Restrictive Health Beliefs Score values.

Differences were also observed according to socio-demographic characteristics. Compared to women, men had lower scores regarding restrictive food beliefs. At the same time, participants with a high school education had higher Restrictive Health Beliefs scores than those with a college education, which was used as the reference category, while participants from rural areas had slightly higher scores than those from urban areas.

The influence of social media was positively associated with the strength of restrictive eating beliefs. Compared to participants who reported the lowest level of social media influence (level 1 on the Likert scale), those who reported higher levels (levels 3–5) had progressively higher Restrictive Health Beliefs scores. In contrast, age and BMI were not significantly associated with the Restrictive Health Beliefs Score.

## 4. Discussion

### 4.1. Restrictive Diets in the Study Sample

Over the last decade, restrictive diets have become increasingly common, including among individuals without medical conditions requiring the elimination of specific foods. In this self-selected online sample, almost one-third of participants (30.9%) reported following a restrictive diet, and 42% of these participants followed the diet without a medical indication. These percentages should not be interpreted as population prevalence estimates because the sampling strategy and demographic composition of the sample do not support extrapolation to the general adult population in Romania. Nevertheless, the findings indicate that restrictive diets without medical indication were reported by a substantial proportion of participants in this nutrition-oriented online sample. This finding suggests that the decision to eliminate certain foods may be associated with factors other than therapeutic necessity.

The demographic composition of the sample, characterized by a high proportion of urban and university-educated participants and a predominance of women, does not reflect the structure of the general adult population in Romania.

Beyond describing the proportion of participants following restrictive diets, the main contribution of this study lies in the separate analysis of medically indicated restrictive diets and those adopted without a medical indication. This distinction revealed different patterns of associations for the two types of dietary behavior, suggesting that the relative importance of the factors associated with restrictive dietary behavior may differ according to whether a medical indication is present or absent.

The percentage observed in this self-selected online sample should not be directly compared with population-based estimates from other countries because of differences in sampling methods and study populations. Nevertheless, studies based on NHANES data and research conducted in Australia provide contextual evidence that restrictive diets, particularly gluten-free diets, are also adopted by individuals without confirmed medical indications [34,35,36,37].

Systematic evidence further indicates that gluten-free, lactose-free, and other “free-from” products are frequently chosen without medical necessity because they are perceived as healthier, more natural, or nutritionally superior [3,4].

Most participants following a restrictive diet reported maintaining it for more than six months, suggesting that this behavior may extend beyond a short-term dietary choice. Repeated food choices may be reinforced by personal experiences and beliefs and may become integrated into an individual’s lifestyle and food identity [38]. In this context, the maintenance of restrictive diets may be associated with both the available nutrition information and individual beliefs about the effects of food on health.

Overall, restrictive diets adopted without medical recommendation represented a relevant dietary behavior among the adults included in this self-selected online sample. These findings support the relevance of examining diet-related health beliefs when seeking to understand restrictive diets adopted without a medical indication.

### 4.2. Restrictive Health Beliefs—A Key Factor Associated with the Adoption of Restrictive Diets

The results of this study suggest that how individuals perceive the relationship between food and health is one of the strongest factors associated with the adoption of a restrictive diet. Participants with higher scores on the Restrictive Health Beliefs scale had higher odds of following both medically justified restrictive diets and restrictive diets adopted without medical recommendation. The higher Restrictive Health Beliefs scores observed in the medically justified diet group should be interpreted in relation to the clinical context, as participants advised to avoid specific foods may reasonably hold stronger beliefs regarding their adverse health effects. However, the significant difference observed between participants without a restrictive diet and those following a restrictive diet without medical indication indicates that the association between Restrictive Health Beliefs and restrictive dietary behavior was not limited to the medically justified group. Moreover, in the case of restrictive diets without medical indication, Restrictive Health Beliefs showed the strongest independent association, suggesting that individual perceptions of the relationship between food and health may be particularly relevant to this dietary behavior. However, because of this study’s cross-sectional design, it cannot be established whether restrictive health beliefs preceded the adoption of restrictive diets or were reinforced after the dietary restriction had already been initiated.

These findings are consistent with health behavior models emphasizing the role of perceived risks and benefits in shaping health-related behaviors [39]. In the case of restrictive diets without medical indication, certain foods may be perceived as health risks and their elimination as beneficial. Thus, dietary behavior may be associated not only with the information available but also with how this information is interpreted through individual health beliefs, as supported by previous research on food-related health perceptions [3,4,27].

Similar patterns have been described in the literature on eating behaviors characterized by excessive concern for healthy eating. Studies on clean eating and orthorexia nervosa similarly describe restrictive eating patterns accompanied by strong beliefs about the beneficial or harmful nature of foods [40,41,42]. In this context, nutrition knowledge and diet-related health beliefs appear to represent distinct dimensions of dietary decision-making, showing different patterns of association with restrictive dietary behavior. Thus, accurate nutritional information alone may not be sufficient to shape dietary behavior when it is interpreted through pre-existing beliefs about diet and health. Similar findings have been reported in studies on nutritional literacy and eating behavior, indicating that accurate information does not necessarily lead to behavioral change when food-related beliefs and attitudes are already well established [43,44,45].

Overall, the results of this study suggest that Restrictive Health Beliefs represent the factor most consistently associated with the adoption of restrictive diets without medical indication. From this perspective, nutrition education interventions should aim not only to improve knowledge about nutrition but also to develop the capacity to critically evaluate information on nutrition and health and to correct beliefs that are not adequately supported by scientific evidence and may be associated with medically unjustified dietary restrictions [46].

### 4.3. Food Vigilance—Between Adaptive Dietary Monitoring and Excessive Concern for Nutrition

The results of the study highlight that the role of food vigilance differs depending on the context in which it occurs, being associated with medically justified restrictive diets but not with those adopted without medical recommendation. Logistic regression analysis showed that Food Vigilance was independently associated only with membership in the medically justified diet group, whereas the association was not statistically significant for restrictive diets without medical indication. These results suggest that careful monitoring of dietary intake may be an adaptive behavior when associated with the need to adhere to dietary treatment. This interpretation is consistent with the literature, which describes food vigilance as an important component of adherence to dietary recommendations in conditions requiring dietary restrictions [3,4]. In contrast, Food Vigilance was not independently associated with restrictive diets adopted without medical indication, despite its association with Restrictive Health Beliefs. This suggests that attention to food alone does not account for voluntary dietary restriction and that its significance may depend on the beliefs about food and health that accompany it. This interpretation is also supported by research on clean eating behaviors and orthorexia nervosa, which shows that an interest in healthy eating is not in itself a problematic behavior [40,47]. However, such concern may become excessive when accompanied by rigid dietary rules and the avoidance of foods perceived as unhealthy without medical justification.

Overall, the results of this study suggest that Food Vigilance should not be automatically interpreted as a risk factor for restrictive eating behaviors. It should be interpreted within the clinical and behavioral context in which it occurs, since it may reflect adaptive behavior when medically justified but is insufficient by itself to explain the adoption of restrictive diets without medical indication.

### 4.4. Nutrition Knowledge Alone Does Not Explain Restrictive Dietary Behavior

Intuitively, it might be assumed that individuals with higher levels of nutrition knowledge are less likely to adopt restrictive diets without medical advice. However, the results of this study did not confirm this hypothesis. Within this specific sample, nutrition knowledge was not independently associated with membership in either restrictive-diet group. This finding should be interpreted in the context of the sample, which consisted predominantly of university-educated participants recruited through online communities focused on nutrition, food intolerances, restrictive diets, and healthy lifestyles. The relatively high and potentially homogeneous Nutrition Knowledge scores may have limited the ability to detect an independent association with diet-group membership. However, participants with higher levels of nutrition knowledge had lower scores on the Restrictive Health Beliefs Scale, suggesting that the relationship between nutrition knowledge and dietary behavior is more complex than a simple direct association. Our results are consistent with the literature, which shows that higher levels of nutrition knowledge are frequently associated with healthier dietary choices, but this relationship is influenced by multiple factors, including individual beliefs, motivation, personal values, social environment, and access to information [43,44,45]. These observations support the view that nutrition education is necessary but may not be sufficient to change eating behavior [43]. Nutrition knowledge can support informed decision-making but does not necessarily translate directly into dietary behavior. Although higher nutrition knowledge was associated with fewer restrictive beliefs about the relationship between nutrition and health, it was not associated with a lower likelihood of following a restrictive diet without medical indication. These results suggest that nutrition knowledge and diet-related health beliefs show different patterns of association with restrictive dietary behavior, although this cross-sectional study cannot establish the direction of these relationships.

Overall, within this specific sample, nutrition knowledge alone did not account for differences in restrictive dietary behavior without medical indication. Dietary decisions may also be associated with how nutritional information is interpreted and integrated into individual health beliefs [46].

### 4.5. The Role of Social Media

The results of this study showed that self-reported social media influence on food choices was not independently associated with restrictive diet-group membership. However, higher reported levels of social media influence were associated with higher Restrictive Health Beliefs scores. This finding should be interpreted cautiously because social media influence was assessed using a single self-reported item. The study did not collect detailed information regarding the reasons for joining nutrition-related online groups, the frequency of exposure, the platforms used, the type of content encountered, or the credibility and professional background of the information sources. Nevertheless, the observed association is consistent with the literature, which describes social media as one of the main sources of nutritional information for young people [48]. Although these platforms facilitate rapid access to nutrition information, they may also facilitate the circulation of simplified messages, recommendations lacking scientific support, and content promoted by influencers without specialized training [46]. In this context, exposure to such content may be associated with beliefs regarding the benefits of eliminating certain foods, even in the absence of strong scientific evidence [49]. Thus, the observed association with Restrictive Health Beliefs but not with restrictive diet-group membership suggests that self-reported social media influence was more clearly associated with diet-related beliefs than with restrictive dietary behavior itself.

These findings may indicate an association between exposure to online nutrition information and diet-related beliefs; however, this study did not test an indirect or mediating pathway between social media use, health beliefs, and restrictive dietary behavior. Moreover, the cross-sectional design does not allow the direction of this association to be established. Online nutrition information may contribute to the formation or reinforcement of diet-related beliefs, but individuals with pre-existing restrictive beliefs may also be more inclined to seek out and engage with such content. Therefore, the findings should not be interpreted as evidence that social media causally shapes restrictive dietary behavior. From a public health perspective, these results highlight the importance of promoting the skills needed to critically evaluate the credibility of nutrition information available online.

### 4.6. Public Health Implications

The results of this study suggest that traditional nutrition education strategies, based primarily on information delivery, may be insufficient to prevent the adoption of restrictive diets without medical indication. Although nutrition knowledge is an essential component of health literacy, our results suggest that dietary decisions are also associated with individual beliefs about the relationship between diet and health. Therefore, public health interventions should combine evidence-based information with critical thinking, assessment of the credibility of information sources, and correction of dietary beliefs that are not supported by scientific evidence. These skills are particularly relevant in the context of rapid access to contradictory nutritional messages [46,48]. The results of the study also highlight the need for the active involvement of health professionals in the communication of evidence-based nutritional information. Collaboration between physicians, dietitians, nutritionists, and public health professionals can contribute to supporting informed dietary decisions.

Taken together, the findings of this study suggest that nutrition education interventions should not be limited to increasing nutrition knowledge alone; they should also promote skills for critically evaluating nutrition information, identifying unsupported claims, and correcting misconceptions about the perceived benefits of eliminating specific foods without medical indication. Such an approach may help reduce the adoption of restrictive diets without a medical indication and promote evidence-based dietary behaviors.

## 5. Limitations and Strengths

The main limitation of the study is related to the online recruitment strategy using convenience sampling. Although this approach facilitated access to people interested in nutrition, health, and restrictive diets, it may have favored respondents already concerned about these topics, thereby introducing a risk of self-selection [23]. Distribution through public posts and reposts prevented the calculation of actual viewing, accessing, or non-participation rates [24,25,26]. The demographic distribution of the sample differed from that of the general adult population in Romania, particularly due to the overrepresentation of urban and university-educated participants. This limits the external validity of the study, and neither the observed percentages nor the magnitude of the associations identified can be directly generalized to the Romanian adult population. Furthermore, the cross-sectional design prevented establishing temporal order or causal relationships between diet-related beliefs, social media use, and restrictive dietary behavior. Social media influence was assessed using a single self-reported item without details on the reasons for joining nutrition-related online groups, frequency of exposure, platforms, content, or source credibility and professional background. Therefore, it remains unclear whether online nutrition information shaped the participants’ beliefs or whether individuals with pre-existing restrictive beliefs selectively sought such content. Furthermore, all information was self-reported, including dietary practices, medical indications, and diagnoses, and was not clinically verified, potentially introducing reporting and classification errors. The questionnaire did not collect free-text details for responses classified as “Other diets”; therefore, the specific dietary patterns included in this heterogeneous category could not be further characterized. The instrument used was developed for the specific objectives of the study and included items selected and adapted from the Health and Taste Attitude Scales [27,28] and the General Nutrition Knowledge Questionnaire/General Nutrition Knowledge Questionnaire-Revised [29,30], together with study-specific items. Accordingly, the composite scores on Restrictive Health Beliefs, Food Vigilance, and Nutrition Knowledge should be considered exploratory indicators requiring further psychometric validation.

The multivariable logistic regression models were considered appropriate for the objectives of the study and the nature of the dependent variable. Although they identified independent associations with the adoption of restrictive diets, they could not simultaneously evaluate more complex structural relationships among variables. Given the cross-sectional design and exploratory nature of the composite measures, structural equation modeling was beyond the scope of this study. Future longitudinal research using fully validated instruments could examine relationships among nutrition knowledge, diet-related health beliefs, food vigilance, and restrictive dietary behavior.

This study was not designed to compare the nutritional adequacy, clinical effectiveness, or health outcomes of specific dietary patterns but to examine the cognitive, behavioral, and socio-demographic factors associated with the adoption of restrictive diets. Accordingly, dietary intake, total energy intake, energy adequacy, macronutrient distribution, protein and fat intake from animal and plant sources, biochemical markers, clinical confirmation of food intolerances or allergies, and eating disorders were not quantitatively evaluated. Weight status was assessed only by BMI calculated from self-reported weight and height; BMI does not distinguish fat mass from lean mass, and no measurements of body fat percentage, waist circumference, or other indicators of body composition were included.

Despite these limitations, the study has several strengths. It addresses an underexplored topic and proposes a classification of participants according to a medical justification for following a restrictive diet, facilitating differentiation between therapeutic dietary restrictions and those adopted voluntarily. It also provides an integrated assessment of socio-demographic factors, nutrition knowledge, diet-related health beliefs, food vigilance, and social media use, providing a more comprehensive examination of voluntary restrictive dietary behavior. The questionnaire was piloted before data collection, which contributed to item clarity and feasibility [31].

## 6. Conclusions

The results of this study suggest that the adoption of restrictive diets without medical indication is more closely associated with beliefs about the relationship between diet and health than with the level of nutrition knowledge. Restrictive Health Beliefs comprised the cognitive factor most strongly associated with this behavior, while Food Vigilance was independently associated only with medically indicated restrictive diets, a pattern that may reflect greater attention to dietary management in a therapeutic context.

The main contribution of this study lies in showing that nutrition knowledge alone did not account for differences in restrictive-diet behavior within this sample, particularly when dietary decisions were associated with strong beliefs about the relationship between diet and health. These findings underscore the importance of distinguishing between nutrition knowledge and diet-related beliefs when examining restrictive dietary behaviors.

From a public health perspective, these findings may inform nutrition education interventions that go beyond the provision of information and include the development of skills for critically evaluating nutrition information and addressing misconceptions about the perceived benefits of restrictive diets adopted without medical indication.

Longitudinal studies using fully validated instruments are needed to confirm these associations, clarify their temporal and potential causal direction, and examine the structural and potential indirect relationships among nutritional knowledge, diet-related health beliefs, food vigilance, and restrictive dietary behavior, including through structural equation modeling where appropriate.

## Figures and Tables

**Table 1:** Sociodemographic and anthropometric characteristics of the participants.

Variable	Category/Indicator	Total (*n* = 766)
Age (years)	Mean ± SD	40.9 ± 11.1
Sex	Female	431 (56.3)
	Male	333 (43.5)
	Prefer not to answer	2 (0.3)
Place of residence	Urban	669 (87.3)
	Rural	97 (12.7)
Education level	High school	103 (13.4)
	College	346 (45.2)
	Master’s degree	284 (37.1)
	Doctorate	33 (4.3)
BMI (kg/m^2^)	Mean ± SD	26.7 ± 5.7

Data are expressed as *n* (%) for categorical variables and as mean ± standard deviation for continuous variables.

**Table 2:** Characteristics of restrictive diets reported by participants.

Variable	Category	*n* (%)
Following a restrictive diet	Yes	237 (30.9)
	No	529 (69.1)
Reported food intolerance	Yes	102 (13.3)
	No	664 (86.7)
Type of intolerance *	Gluten	76 (74.5%)
	Lactose	57 (55.9%)
	Histamine	14 (13.7%)
Classification of participants	No restrictive diet	529 (69.1)
	Medically justified diet	137 (17.9)
	Diet without medical indication	100 (13.1)
Types of restrictive diets **	Gluten-free	101 (42.6)
	Lactose-free	65 (27.4)
	Low-histamine	7 (3)
	Iodine-free	7 (3)
	Alkaline diet	3 (1.3)
	Other diets	117 (49.4)
Duration of diet	<1 month	31 (13.2)
	1–3 months	33 (14.0)
	3–6 months	33 (14.0)
	>6 months	138 (58.7)

* Percentages were calculated among participants who reported a food intolerance (*n* = 102). Participants could report more than one type of intolerance; therefore, categories were not mutually exclusive, and percentages may exceed 100%. ** Percentages were calculated among participants who reported following a restrictive diet (*n* = 237). Participants could select more than one type of restrictive diet; therefore, categories were not mutually exclusive, and percentages may exceed 100%. The “Other diets” category included diets not covered by the predefined response options and was not further specified in the questionnaire. Duration data were available for 235 of the 237 participants who reported following a restrictive diet.

**Table 3:** Descriptive statistics and internal consistency of composite scores.

Score	Mean ± SD	Median	Minimum	Maximum	Cronbach’s α
Restrictive Health Beliefs Score	1.84 ± 0.84	1.75	0.88	4.75	0.852
Food Vigilance Score	3.46 ± 0.67	3.56	1.00	5.00	0.758
Nutrition Knowledge Score	14.2 ± 1.77	15.0	6	17	-

Cronbach’s α was calculated for scores derived from Likert-type items. The Nutrition Knowledge Score was calculated by summing the correct responses and was not assessed for internal consistency.

**Table 4:** Comparison of composite scores among the three groups of participants.

Variable	No Restrictive Diet (*n* = 529) Md (IQR)	Medically Indicated Diet (*n* = 137) Md (IQR)	Diet Without Medical Indication (*n* = 100) Md (IQR)	χ^2^	*p*	ε^2^
Restrictive Health Beliefs Score	1.38 (1.13)	2.50 (1.25)	2.00 (1.50)	100.72	<0.001	0.132
Food Vigilance Score	3.33 (0.89)	4.00 (0.89)	3.56 (1.03)	70.74	<0.001	0.092
Nutrition Knowledge Score	15 (2)	14 (2)	15 (3)	2.45	0.293	0.003

Values are presented as medians (IQRs). Differences between groups were assessed using the Kruskal–Wallis test. ε^2^ represents the effect size.

**Table 5:** Characteristics of participants by diet group.

Variable	Medically Indicated Diet (*n* = 137)	Diet Without Medical Indication (*n* = 100)	No Restrictive Diet (*n* = 529)	*p*
Sex, *n* (%)				<0.001
Women, *n* (%)	117 (85.4)	61 (61.0)	253 (47.8)	
Male, *n* (%)	20 (14.6)	39 (39.0)	274 (51.8)	
Prefer not to answer, *n* (%)	0 (0.0)	0 (0.0)	2 (0.4)	
Urban, *n* (%)	108 (78.8)	92 (92.0)	469 (88.7)	0.003
Age, years (median)	41	43	41	0.542
BMI, median	24.8	26.2	26.2	0.014
College education, (%)	93.1	81.0	83.6	0.176

Data are presented as *n* (%) for categorical variables and as median for continuous variables. The *p*-value for sex refers to the overall comparison of the distribution of sex categories across the three diet groups.

**Table 6:** Spearman correlations between composite scores and analyzed variables.

Variable	ρ	*p*
Restrictive Health Beliefs–Food Vigilance	0.378	<0.001
Restrictive Health Beliefs–Nutrition Knowledge	−0.264	<0.001
Restrictive Health Beliefs–BMI	−0.077	0.034
Food Vigilance–Age	0.133	<0.001
Food Vigilance–BMI	−0.097	0.007
Social media–Restrictive Health Beliefs	0.231	<0.001

**Table 7:** Factors associated with diet group membership (reference category: no restrictive diet).

Predictor	Medically Indicated Diet OR (95% CI)	*p*	Diet Without Medical Indication OR (95% CI)	*p*
Male sex	0.19 (0.11–0.33)	<0.001	0.65 (0.41–1.03)	0.067
Restrictive Health Beliefs Score	2.79 (2.05–3.80)	<0.001	1.68 (1.24–2.28)	<0.001
Food Vigilance Score	2.25 (1.51–3.34)	<0.001	1.08 (0.74–1.58)	0.682
Nutrition Knowledge Score	1.06 (0.93–1.21)	0.402	1.01 (0.89–1.16)	0.839
Social media (global)	-	0.091	-	0.091
BMI	0.98 (0.94–1.02)	0.247	1.00 (0.96–1.04)	0.874
Age	1.00 (0.98–1.02)	0.805	1.01 (0.99–1.03)	0.340

OR = odds ratio; 95% CI = 95% confidence interval. Participants without a restrictive diet were used as the reference category for the outcome, and female sex was used as the reference category for sex. For the social media variable, the omnibus test of the predictor in the multinomial model is reported.

**Table 8:** Factors independently associated with Restrictive Health Beliefs Score.

Predictor	B	*p*
Food Vigilance Score	0.457	<0.001
Nutrition Knowledge Score	−0.111	<0.001
Male (vs. female)	−0.122	0.029
Urban (vs. rural)	−0.167	0.037
High school (vs. College)	0.279	<0.001
Master’s degree (vs. College)	−0.005	0.925
Doctorate (vs. College)	0.153	0.246
Social media: score 3 vs. 1	0.170	0.014
Social media: score 4 vs. 1	0.411	<0.001
Social media: score 5 vs. 1	0.375	0.002
BMI	0.001	0.780
Age	−0.002	0.559

The model was statistically significant (F = 21.5; *p* < 0.001) and explained 28.6% of the variance in the Restrictive Health Beliefs Score (R^2^ = 0.286).

## Data Availability

The data are not publicly available due to privacy and ethical restrictions. Aggregated results are available from the corresponding author upon reasonable request.

## Supplementary Materials

The following supporting information can be downloaded at: https://www.mdpi.com/article/10.3390/nu18162685/s1. The full questionnaire used in the study is provided in English as Supplementary File S1: Restrictive_Diets_Questionnaire. Supplementary File S2: STROBE Checklist for Cross-Sectional Studies [22].

## Abbreviations

The following abbreviations are used in this manuscript:

BMI	Body Mass Index
CI	Confidence Interval
GNKQ	General Nutrition Knowledge Questionnaire
GNKQ-R	General Nutrition Knowledge Questionnaire-Revised
HTAS	Health and Taste Attitude Scales
IQR	Interquartile Range
OR	Odds Ratio
SD	Standard Deviation
WHO	World Health Organization
