# Supplementary material for: Association of Diet- and Health-Related Beliefs with Restrictive Diets Without Medical Indication in a Self-Selected Online Sample of Adults in Romania: A Cross-Sectional Study
Source: Nutrients. 2026 Aug 17;18(16):2685. doi: 10.3390/nu18162685 (PMC13517075; doi:10.3390/nu18162685)
Supplement: Supplementary file 1 [file nutrients-18-02685-s001.zip › Supplementary File S2_STROBE checklist.pdf]

Supplementary File S2. STROBE Checklist for Cross-Sectional Studies

Manuscript: Association of Diet- and Health-Related Beliefs with Restrictive Diets Without Medical Indication in a Self-Selected Online Sample of Adults in Romania: A Cross-Sectional Study

This checklist maps the reporting items of the STROBE Statement for cross-sectional studies to the corresponding pages and sections of the manuscript. Reporting-item descriptions are concise paraphrases of the STROBE checklist.

Section	Item no.	Reporting recommendation	Page	Location in manuscript
Title and abstract	1(a)	Identify the study design in the title or abstract.	1	Title
	1(b)	Provide an informative, balanced abstract summarizing methods and main findings.	2	Abstract
Introduction	2	Explain the scientific background and rationale for the investigation.	2–4	Introduction
	3	State the specific objectives and any prespecified hypotheses.	4	End of Introduction
Methods	4	Present the key elements of the study design early in the Methods section.	4	Section 2.1
	5	Describe setting, location, recruitment context, and relevant dates.	4–5	Sections 2.1–2.2
	6	Report eligibility criteria and the sources and methods used to select participants.	4–5	Sections 2.1–2.2
	7	Define the main outcomes, exposures/predictors, potential confounders, and diagnostic/classification criteria.	7–9	Sections 2.4–2.6
	8	Describe data sources and measurement methods for variables of interest and their comparability across groups.	6–9	Sections 2.3–2.6
	9	Describe measures taken to address potential sources of bias.	6; 21–22	Section 2.2; Section 5
	10	Explain how the study size was determined.	4	Section 2.1
	11	Explain how quantitative variables were handled in the analyses and any categorization used.	7–9	Sections 2.5–2.7
	12(a)	Describe the statistical methods, including methods used to account for confounding.	9–10	Section 2.7
	12(b)	Describe methods used for subgroup analyses or interactions, when applicable.	9	Section 2.7
	12(c)	Explain how missing data were handled.	10	Section 2.7

	12(d)	Describe analytical methods that account for the sampling strategy, when applicable.	N/A	Convenience online sample; no survey weighting/design-based analysis
	12(e)	Describe sensitivity analyses, when performed.	N/A	No sensitivity analyses were performed
Results	13(a)	Report participant numbers at the relevant stages of recruitment, inclusion, and analysis.	5–6	Section 2.2; Figure 1
	13(b)	Give reasons for exclusions or non-participation where known.	5	Section 2.2
	13(c)	Use a flow diagram when useful.	6	Figure 1
	14(a)	Report participant characteristics and relevant exposure/confounder information.	10–13	Sections 3.1 and 3.5; Tables 1 and 5
	14(b)	Indicate the number of participants with missing data for variables of interest.	10–11	Section 2.7; Table 2 footnote
	15	Report outcome/event numbers or appropriate summary measures.	11–12	Sections 3.2–3.3; Tables 2–3
	16(a)	Report main estimates with precision and clarify the variables included in adjusted models.	14–15	Section 3.7; Table 7
	16(b)	Report category boundaries when continuous variables are categorized.	7–8	Section 2.5
	16(c)	Translate relative estimates into absolute risk over a meaningful period when relevant.	N/A	Not applicable to this cross-sectional analysis
	17	Report other analyses, such as subgroup, interaction, or sensitivity analyses, when performed.	12–16	Sections 3.4–3.8; no sensitivity analyses
Discussion	18	Summarize the key findings in relation to the study objectives.	16–22	Sections 4–6
	19	Discuss study limitations, including potential bias and imprecision and their likely implications.	21–22	Section 5
	20	Provide a cautious overall interpretation considering objectives, limitations, multiple analyses, and related evidence.	16–22	Sections 4–6
	21	Discuss the generalizability (external validity) of the findings.	21–22	Section 5
Other information	22	Report the funding source and the role of funders, if any.	25	Funding statement

Strobe reference: von Elm, E.; Altman, D.G.; Egger, M.; Pocock, S.J.; Gøtzsche, P.C.; Vandenbroucke, J.P.; for the STROBE Initiative The Strengthening the Reporting of Observational Studies in Epidemiology (STROBE) Statement: Guidelines for Reporting Observational Studies. Ann Intern Med 2007, 147, 573–577, doi:10.7326/0003-4819-147-8-200710160-00010
