# Supplementary material for: Association of Diet- and Health-Related Beliefs with Restrictive Diets Without Medical Indication in a Self-Selected Online Sample of Adults in Romania: A Cross-Sectional Study
Source: Nutrients. 2026 Aug 17;18(16):2685. doi: 10.3390/nu18162685 (PMC13517075; doi:10.3390/nu18162685)
Supplement: Supplementary file 1 [file nutrients-18-02685-s001.zip › Supplementary File S1. Restrictive_Diets_Questionnaire.pdf]

Supplementary File S1. Restrictive_Diets_Questionnaire

You are invited to participate in a research study by completing this questionnaire. The aim of this study is to assess the tendency to adopt restrictive diets without a medical indication among adults in Romania.

This study is being conducted within the Faculty of Medicine, Iuliu Hațieganu University of Medicine and Pharmacy, Cluj-Napoca.

Your responses will contribute to a better understanding of the motivations and potential risks associated with following restrictive diets without medical indication, as well as to the development of appropriate preventive strategies. Your participation will provide valuable information regarding these risk factors. The findings of this study will be published in the scientific literature and may be consulted and used by healthcare professionals as well as patients. This online questionnaire is anonymous. To ensure confidentiality, please do not provide any personally identifiable information (such as your full name, address, telephone number, or email address).Participation in this study is entirely voluntary, and your responses cannot be linked to your identity. By completing this questionnaire, you consent to participate in this study. Please answer each question by selecting the appropriate response or by providing the requested information where applicable. We kindly ask that you answer all questions as honestly as possible.

Thank you for participating in this research project and for taking the time to complete this questionnaire.

I agree to participate in this study. (Required response)

☐ Yes

Section 1 – Socio-demographic Information

1. Age (years)
2. Gender – Male / Female / Other / Prefer not to answer
3. Place of origin – Urban / Rural
4. Level of education – High school / Bachelor's degree / Master's degree / Doctorate
5. Height ______ / Weight ______

Section 2 – Dietary Habits and Restrictive Diets

1. Are you currently following a special diet? – Yes / No
2. If yes, which one? Gluten-free/ Lactose-free/ Low-histamine/ Iodized salt-free (or iodine-free)/ Alkaline diet/ Other diet/ I am not following any diet
3. How long have you been following this diet?- Less than 1 month/ 1–3 months/ 3–6 months/ More than 6 months
4. Was this diet recommended by a healthcare professional? – Yes / No
5. Have you ever been diagnosed with an intolerance to gluten, lactose, or histamine? – Yes / No / I don't know
6. If yes, which one?

Section 3 – Motivations and Perceptions

Instruction: Please indicate your level of agreement with each statement using the Likert scale:

1 = Strongly disagree; 5 = Strongly agree

1. I believe that a gluten-free, lactose-free, low-histamine, iodized salt-free, or low-acid diet is healthier.
2. I chose this diet to prevent digestive problems.
3. I believe that gluten-free, lactose-free, low-histamine, iodine-free, or low-acid foods are more natural.
4. I believe that foods containing gluten are harmful.
5. I believe that foods containing lactose are harmful.
6. I believe that foods high in histamine (such as aged or fermented dairy products, canned foods, fish, tomatoes, legumes, etc.) are harmful.
7. I believe that iodized salt is harmful.
8. I believe that acidic foods (e.g., meat, milk, cheese, butter, soy products, etc.) are harmful.

9. I rely on social media when making food choices.
10. I believe that the diet I follow reduces inflammation in the body.

Section 4 – Attitudes Toward Healthy Eating and Food Vigilance

Instruction: Please indicate your level of agreement with each statement.
1 = Strongly disagree; 5 = Strongly agree

1. It is important to me that my daily diet is healthy.
2. I choose food products based on their nutritional value.
3. I prefer natural foods, even if they are more expensive.
4. I enjoy trying new and natural food products.
5. Taste is less important than health benefits.
6. I read food labels to avoid certain ingredients (e.g., gluten or lactose).
7. I avoid products containing additives or preservatives.
8. I feel guilty when I eat "unhealthy" foods.

Section 5 – Nutrition Knowledge

1. How many servings of fruits and vegetables are recommended per day?- 2/ 3/ 5/ 8
2. What is the primary source of dietary fiber?- Meat/ Fruits and vegetables/ Cheese/ Eggs/
3. What is the recommended daily salt intake for a healthy adult?- 2 g/ 5 g/ 10 g/ 15 g
4. Which food contains the highest amount of calcium?- Milk/ Apples/ Bread/ Eggs
5. Which of the following foods is highest in saturated fat? - Butter/ Avocado/ Nuts/ Olive oil
6. Which food contains the highest amount of protein? – Chicken / Potatoes / Bread / Bananas
7. Excessive sugar consumption is associated with diabetes. – True / False
8. A diet high in fiber reduces the risk of constipation and cardiovascular disease. – True / False
9. Which beverage is the best choice for daily hydration? – Coffee / Water / Sweetened beverages / Energy drink / Tea / Other (e.g., clear soups, freshly squeezed juices)
10. Which of the following snacks is the healthiest? – Breakfast cereal with milk/ Fresh fruit / Chocolate bar / Sweet biscuits / Chips / Other
11. Which type of fat is considered more beneficial for heart health?- – Saturated fats (found in milk, butter, cream, lard, fatty meat, palm oil, coconut oil) / Unsaturated fats (nuts, olives, avocado, fish, vegetable oils) / Both / Neither
12. Gluten is harmful to everyone. – True / False
13. Dairy products should be eliminated to lose weight. – True / False
14. A gluten-free diet is automatically healthier. – True / False
15. People without an intolerance can safely consume milk and dairy products. – True / False
16. Eliminating certain food groups without medical advice may lead to nutritional deficiencies. – True / False
17. A balanced diet includes foods from all major food groups. – True / False

Section 6 – Preventive Behaviors

1. Did you consult a physician or a registered dietitian before starting this diet? – Yes / No
2. Would you recommend a gluten-free or lactose-free diet to others without prior medical testing? – Yes / No
